# Soil Inoculation with *Bacillus* spp. Modifies Root Endophytic Bacterial Diversity, Evenness, and Community Composition in a Context-Specific Manner

**DOI:** 10.1007/s00248-018-1160-x

**Published:** 2018-03-06

**Authors:** Kiran R. Gadhave, Paul F. Devlin, Andreas Ebertz, Arabella Ross, Alan C. Gange

**Affiliations:** 1 School of Biological Sciences, Royal Holloway University of London, Egham, Surrey TW20 0EX UK; 2 Department of Entomology and Plant Pathology, North Carolina State University, Raleigh, NC USA; 3 Centre for Systems and Synthetic Biology, Royal Holloway University of London, Egham, Surrey TW20 0EX UK

**Keywords:** *Bacillus*, Plant growth-promoting rhizobacteria, Endophytes, 454 pyrosequencing, Microbial inoculants

## Abstract

The use of microbial inoculants containing plant growth-promoting rhizobacteria as a promoter of plant fitness and health is becoming increasingly popular in agriculture. However, whether and how these bacteria affect indigenous bacterial communities in field conditions is sparsely explored. We studied the effects of seed inoculation and field soil application of ubiquitous soil bacteria, *B*. *cereus*, *B*. *subtilis*, and *B*. *amyloliquefaciens*, on the diversity, evenness, and richness of endophytic bacterial communities in sprouting broccoli roots using high-throughput metagenome sequencing. The multiple operational taxonomic units (OTUs) assigned to different bacterial taxa clearly showed changes in ecological measures and relative abundances of certain taxa between control and treatment groups. The *Bacillus* inocula, themselves, failed to flourish as endophytes; however, the effects they extended on the endophytic bacterial community were both generic as well as species specific. In each case, *Pseudomonadales*, *Rhizobiales*, *Xanthomonadales*, and *Burkholderiales* were the most abundant orders in the endosphere. *B*. *amyloliquefaciens* drastically reduced the most abundant genus, *Pseudomonas*, while increasing the relative abundance of a range of minor taxa. The Shannon-Weiner diversity and Buzas and Gibson’s evenness indices showed that the diversity and evenness were increased in both *B*. *amyloliquefaciens* and mixed treated plants. The UniFrac measurement of beta diversity showed that all treatments affected the specific composition of the endophytic bacterial community, with an apparent interspecies competition in the mixed treatment. Taken together, *Bacillus* species influenced the diversity, evenness, and composition of the endophytic bacterial community. However, these effects varied between different *Bacillus* spp. in a context-specific manner.

## Introduction

The bacterial communities of the plant microbiome can have a wide range of beneficial effects on plant growth and on pest and disease resistance [1]. However, such communities may be impoverished in agricultural systems, leading to the development of microbial inoculants that aim to provide similar benefits [2, 3]. The use of such bacterial inoculants, as a sustainable alternative to agrochemicals, has been widely studied in recent years. There is a growing amount of evidence that plant growth-promoting rhizobacteria (PGPR) can be effectively used to increase plant health and productivity, suppress plant diseases and pests, and mitigate the effects of abiotic stresses [4]. However, while PGPR can exert considerable effects in their own right, it is likely that they will also influence the composition of the internal microbiome, or endosphere [5]. Since bacterial endophytes are ubiquitous in most plant species and provide “natural” plant health and fitness benefits [6–8], it is critical to understand whether and how the addition of common species of PGPR, as inoculants, influences the indigenous root endophytic bacterial community.

There seems to be a dispute in the literature as to whether endophytic bacteria are simply subsets of the rhizoplane and rhizosphere bacterial community. For instance, a few early studies suggest that endophytes are recruited by plants from the rhizoplane community [9–11], while more recent ones suggest that, despite some overlapping species, root endophytic communities are distinct from those that exist in rhizosphere [12, 13]. It appears that a variety of factors, plant species, genotype, physiological stage, microbial competence for nutrients and niche, and soil type, play important roles in shaping the endophytic community structure, function, and dynamics [5, 14]. The effects of only a small number of external bacterial inocula on the endosphere have been tested. For instance, Conn and Franco [14], using terminal restriction fragment length polymorphism (T-RFLP), showed that colonization of wheat roots by a non-adapted mixed actinobacterial inoculum disturbed the natural actinobacterial endophyte population through severe reductions in diversity and colonization levels, whereas the addition of a single indigenous actinobacterial species increased its colonization level without any detriment to the indigenous endophyte population. Schmidt et al. [15] showed that the microbiome of chamomile, *Chamomilla recutita*, when analyzed using pyrosequencing, showed a shift within the bacterial community structure and beta diversity indices between control plants and those inoculated with a variety of (mostly non-*Bacillus*) bacteria.

Despite the fact that *Bacillus* is one of the most commonly used genera in microbial inocula, no studies have looked at their effect as an inoculum on the endospheric bacterial community. *Bacillus* is the predominant rhizosphere genus [16] and one of the most commonly isolated endorhizal genera [7, 17]. Several studies have reported that the principal plant-associated bacteria, *B*. *cereus*, *B*. *subtilis*, and *B*. *amyloliquefaciens*, increase nutrient availability, vegetative growth, flower quality, reproductive maturity, and resistance to plant pathogens, pests, and edaphic factors [18–23]. Earlier studies showed that all three species of *Bacillus* are persistent root colonizers of diverse plants and exist as endophytes throughout most plant growth stages (e.g., *B*. *cereus* [24]; *B*. *subtilis* [25]; *B*. *amyloliquefaciens* [26]). One very recent study has examined the impact of *B*. *subtilis* supplementation on the rhizosphere bacterial community [27]. This study found only a very short-lived effect of *B*. *subtilis* supplementation on the rhizospheric bacterial community, contrary to the longer term benefits observed in crops grown in supplemented soil. In this case, bacterial supplementation only affected the tomato rhizosphere bacterial community for 3 days. However, that study did not examine the effects on the endospheric bacterial population [27]. Given all that, it is important to determine the effects of all of these widely adopted inoculants containing *Bacillus* spp. on indigenous microbial communities, so their applicability in the field can be effectively tested.

We studied the effects of individual and mixed application of *B*. *cereus*, *B*. *subtilis*, and *B*. *amyloliquefaciens* on diversity, evenness, abundance, and richness of the endorhizal bacterial community in sprouting broccoli (*Brassica oleracea* L.) using the 454 pyrosequencing. We chose this plant as a model crop as this experiment was part of a wider study investigating application of soil inoculants on foliar-feeding insects [28]. We hypothesized that the additions of ubiquitous *Bacillus* species to sprouting broccoli will alter the attributes of the endophytic bacterial community, but this will depend on the identity of the species added, since different species affect plants in different ways [29]. A better understanding of the consequence of *Bacillus* inoculant addition is urgently required if these products are to become consistent in their effects, and so realize their full potential in sustainable agriculture [30].

## Materials and Methods

### Bacterial Inoculation, Sowing, and Aftercare

Before sowing, 450 seeds of autumn broccoli cv. Green sprouting broccoli (Country Value Seeds, UK) were placed in 50-ml falcon tube and surface sterilized with 40 ml of a 2% sodium hypochlorite by vigorous shaking for 20 min. Following sterilization, seeds were washed five times with sterile distilled water and decanted on to a Petri plate in a laminar hood. The bacterial species *B*. *cereus* no. 8 FW Athal, *B*. *subtilis* NRRLB23051, and *B*. *amyloliquefaciens* subsp. *plantarum* FZB42-BGSC10A6 were obtained from Dr. B. Raymond (Imperial College London, UK). All three bacterial species were originally isolated from the roots of *Arabidopsis thaliana*, a model plant from the same plant family (Brassicaceae) as sprouting broccoli, and were cryopreserved at − 80 °C in 80% (*v*/*v*) glycerol stock. These cryopreserved bacterial cultures were recovered on 20 ml LB broth and incubated at 37 °C overnight. The tubes containing cultures were serially diluted in 0.85% saline water and 50 μl of a 10^−5^ dilution of each bacterium was plated on LB agar medium individually to determine colony-forming units ml^−1^. The sterilized seeds were arranged in five squared 150 × 20 mm Petri plates, with 90 seeds per plate and one of five treatments applied to each; control (seeds imbibed for 3 h with 210 ml sterile distilled water); individual treatments of 210 ml of 10^8^ cfu ml^−1^ suspensions of *B*. *cereus*, *B*. *subtilis*, and *B*. *amyloliquefaciens* and 210 ml, 10^8^ cfu ml^−1^ mixture of 70 ml of each of these bacteria.

The field experiment was carried out at Royal Holloway, University of London, Egham, Surrey, on a plot measuring 20 × 10 m, from June to September 2014. The plot was laid out in five blocks, each 4 × 2 m and containing five planting rows. Each row in the block was planted with three randomly picked seeds per station, 30 cm apart, with the aim of retaining one vigorous seeding after thinning. Seeds were planted out in a randomized block design with five rows in each block. Eventually, 30 replicate plants (6 per block, 5 blocks) per each treatment were maintained throughout the experiment. One supplementary 210-ml addition was applied through drenching slowly at the base of each plant of the respective treatments as described above 3 weeks after sowing, to ensure bacterial colonization. Plants were grown in organic conditions, without the applications of pesticides and fertilizers. Plants were irrigated with water once every 2 days and weeded twice at an interval of 20 days.

### Total Bacterial DNA Extraction

To extract total bacterial DNA from roots, four plants per treatment were sampled approximately 10 cm below soil surface randomly at 30 days after sowing. The DNA extraction from each root sample was performed immediately after harvesting following the modified SDS-based procedure of Zhou et al. [31]. Prior to DNA extraction, roots were washed under tap water, drained to remove excess water, weighed, and transferred to 50-ml falcon tubes. Surface sterilization of the roots was carried out using 30 ml, 70% ethanol followed by 10% sodium hypochlorite, with continuous vortexing for 5 min. The traces of these chemicals were removed by washing roots with sterile distilled water five times, with intermittent vortexing for 1 min. The 100 μl water from the fifth wash was plated on LB media plates, which were incubated at 37 °C for 3 days to confirm that the surface sterilization was successful. The 3-g roots from each sample were ground in liquid nitrogen using a sterilized mortar and pestle.

The 13.5 ml of DNA extraction buffer [100 mM Tris-HCl (pH 8.0), 100 mM sodium EDTA (pH 8.0), 100 mM sodium phosphate (pH 8.0), 1.5 M NaCl, 1 CTAB] and 100 μl of proteinase K (10 mg ml^−1^) were added to 30-ml Oakridge high-speed centrifuge tubes containing surface sterilized broken up roots. The contents were mixed by rotary shaking at 225 rpm for 30 min at 37 °C. The 1.5 ml of 5% SDS was added, and the tubes were incubated in a 65 °C water bath for 2 h with intermittent gentle inversions every 15 to 20 min. The contents were centrifuged at 6000×*g* for 10 min at room temperature, and the collected supernatants were transferred into 50-ml centrifuge tubes. The extraction procedure was repeated twice by adding 4.5 ml of the DNA extraction buffer and 0.5 ml of 5% SDS to the root tissue, vortexing, incubating at 65 °C for 10 min, and centrifuging as specified. The supernatants from the three extractions were combined and mixed with an equal volume of chloroform: isoamyl alcohol (24:1, *v*/*v*). The extracts were centrifuged, and the aqueous phase was recovered and precipitated with 0.6 volume of isopropanol at room temperature for 1 h. The crude DNA pellet was obtained through centrifugation at 16000×*g* for 20 min at room temperature and was later washed with cold 70% ethanol and resuspended with sterile deionized water to make 500-μl total bacterial DNA suspension. The DNA suspensions were transferred to 2-ml Eppendorf tubes, quantified using NanoDrop, and stored at − 20 °C until further use.

### Amplification and High-Throughput Sequencing of 16S rRNA Amplicons

For each treatment, the four PCR-amplified replicates containing appropriate DNA quantities based on standardization (approx. 100 ng μl^−1^) were sequenced as a pool so as to minimize individual inter-plant differences. The universal primers, U515F (59-TGYCAGCMGCCGCGGTA) and U927R (59-CCCGYCAATTCMTTTRAGT), were designed to amplify the V4 and V5 regions of the 16S rRNA gene from the extracted total bacterial DNA samples to get the best possible taxonomic resolution. The forward fusion primers contained the GS FLX Titanium primer A, the library key (59-CCATCTCATCCCTGCGTGTCTCCGACTCAG), and one 10 base multiplex identifier (MID) (Roche Diagnostics Ltd., UK), whereas the reverse fusion primers included the GS FLX Titanium primer B and the library key (59-CCTATCCCCTGTGTGCCTTGGCAGTCTCAG). To avoid misidentification at the demultiplexing stage, at least two base differences between MIDs were maintained. The following cycling conditions were followed for amplification, which was performed using FastStart HiFi Polymerase (Roche Diagnostics Ltd., UK): 94 °C for 3 min; 30 cycles of 94 °C for 30 s, 55 °C for 45 s, 72 °C for 1 min; followed by 72 °C for 8 min with 30 cycles. The Ampure XP magnetic beads (Beckman Coulter) were used to purify amplicons and the fluorescence-based Picogreen assay (Invitrogen) was performed to measure the concentration of each sample. The unique MIDs were used to identify each normalized sample and unidirectional sequencing from forward primers was performed in separate picotiter plate regions on the GS FLX Titanium platform following manufacturer’s guidelines (Roche Diagnostics, UK).

### Data Analyses

The data were processed using the Quantitative Insights Into Microbial Ecology software package (QIIME v1.8.0). The dataset was split into each separate file per sample according to MID adapters using “Ampliconnoise” pipeline and the pyrosequencing, PCR, and chimeric errors were removed. The cleaned sequences containing at least 400 bases were used to produce OTUs, with 97% similarity cutoff, which were further aligned and clustered using “Pick_de_novo_otus” pipeline. For each sample, the number of operational taxonomic units (OTUs) allocated and percent relative abundances were determined at multiple taxonomic levels. Plant chloroplast and mitochondria sequences were removed using the “Filter_otus_from_otu_table” command.

The ecological diversity measures Shannon-Weiner diversity index and Buzas and Gibson’s species evenness [32] were used to analyze bacterial communities in each treatment group. These indices were calculated using the PAST3 software suite [32] following random rarefaction of all samples to the same number of individuals as present in the smallest sample. Statistical significance of differences between control and treated samples was calculated via the PAST3 Diversity *t* test function [33].

The principal coordinate analysis (PCoA) plots for beta diversity, displaying jackknifed confidence ellipses were plotted using the “Jackknifed_beta_diversity” pipeline to represent the variation in the microbial community between different treatments. Here, beta diversity was represented using the weighted (quantitative) UniFrac distance measure [34], which is a phylogenetically aware measure of beta diversity. Calculation of UniFrac beta diversity uses information stored in a Newick format phylogenetic tree generated by the “Pick_de_novo_otus” pipeline which was re-run following removal of plant chloroplast and mitochondria sequences via the “Filter_fasta” command. We generated ten jackknife rarefied OTU table replicates based on randomly chosen subsamples from each set of sequences equal in size to 75% of the smallest sample. As part of the pipeline, UniFrac distance matrices are then generated from rarefied OTU tables and PCoA computed for each, producing the 95% confidence ellipsoids plotted.

## Results

We sought to analyze the effect of common *Bacillus* supplementation treatments on the endophytic bacterial communities of field-grown sprouting broccoli. Various single and mixed *Bacillus* inocula were tested via an initial seed surface inoculation followed by a subsequent addition to soil shortly after germination. De-noised and filtered metagenomic sequencing data from each treatment was analyzed using QIIME to generate taxa abundance profiles at several taxonomic levels. The reads showing more than 97% sequence homology revealed a very high proportion of plant chloroplast and mitochondria sequences, a common problem in analysis of endophytic bacteria due to the relatedness of chloroplast and mitochondria ribosomal RNA-encoding sequences to those of bacteria. Nonetheless, 1817 sequences corresponding to endophytic bacteria were assigned to 14 phyla, 26 classes, 50 orders, 91 families, and 231 genera. Although sequencing depth was low, with most genera represented by only one or two individuals and showing little co-occurrence across treatment groups, consistent patterns were revealed when analyzing more abundant genera.

**Fig. 1:**
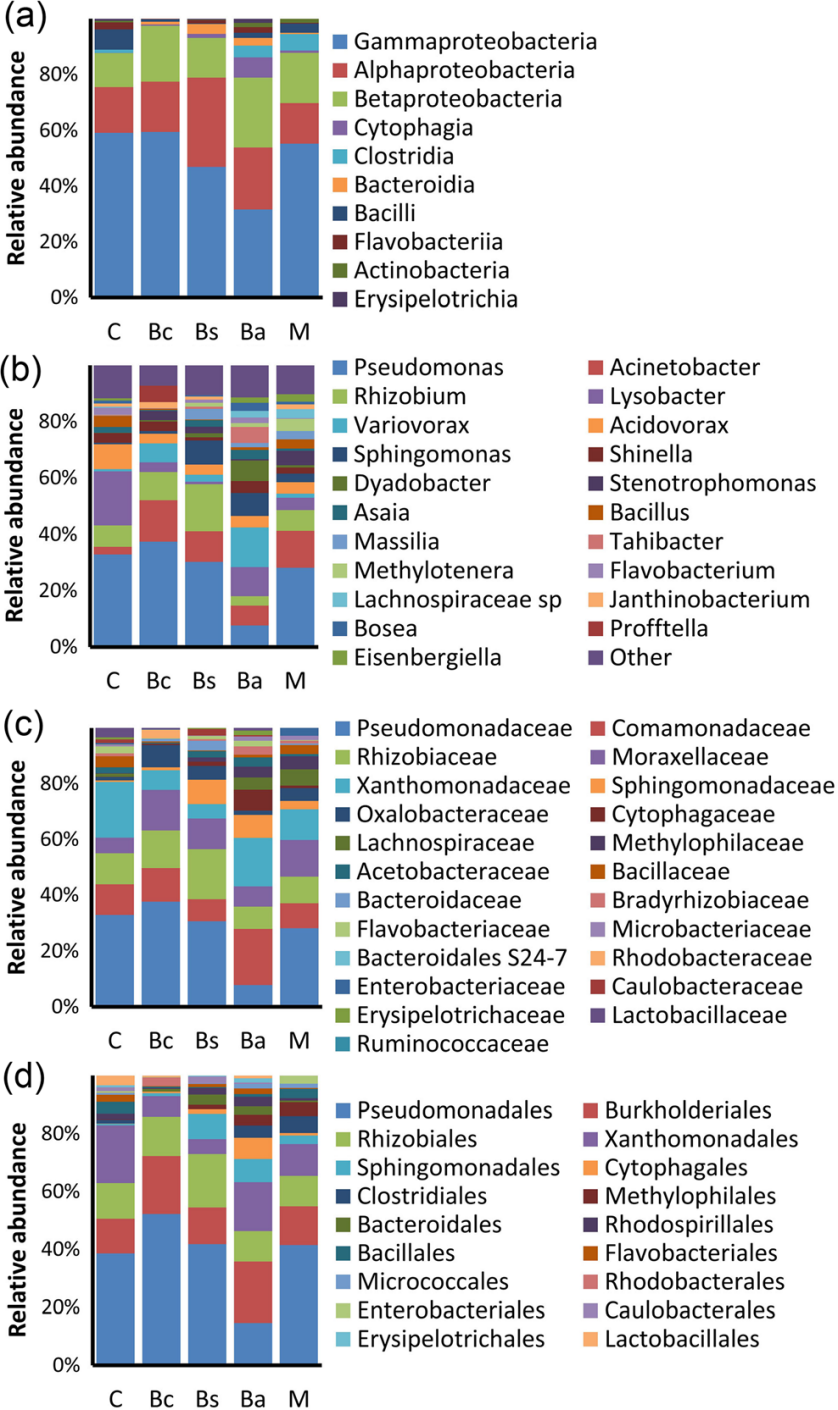
Comparison of the most abundant taxa within the sprouting broccoli endosphere. The notations B. c., B. s., B. a. represent *B*. *cereus*, *B*. *subtilis*, and *B*. *amyloliquefaciens* respectively. **a** Class. **b** Genus. **c** Family. **d** Order. Genera contributing ≥ 2% of population (after removing chloroplast, mitochondria, and unclassified sequences)

Comparison of the abundances of taxa contributing greater than or equal to 2% of the classified endophytic population revealed a high degree of commonality in composition of the endophytic community from both the control and treated samples. The vast majority of all communities consisted of Alpha-, Beta-, and Gamma-proteobacteria, in keeping with previous observations for endophyte communities in a range of plant species [35] (Fig. 1a). Curiously, all samples treated with *Bacillus* inocula showed a significant reduction in the relative abundance of Bacilli recovered in the endophyte community, perhaps suggesting some antagonism between the externally introduced and endophytic Bacilli. Other than that difference, only the *B*. *amyloliquefaciens*-treated sample showed any marked distinction from the control at the class level. The *B*. *amyloliquefaciens*-treated sample showed a prominent drop in the relative abundance of Gammaproteobacteria as well as a higher relative abundance of non-*Proteobacteria*, with a notable occurrence of *Cytophagia*; though, neither of these changes were observed in the mixed sample, which also contained *B*. *amyloliquefaciens* (Fig. 1a). Examination at the level of genus reveals this reduction in Gammaproteobacteria in the *B*. *amyloliquefaciens*-treated sample to be largely accounted for by a marked reduction in the relative abundance of *Pseudomonas*. In all other samples, *Pseudomonas* formed the most abundant genus, accounting for approximately 30% of the identified endophytic bacteria whereas *Pseudomonas* made up only 7% of the community of the *B*. *amyloliquefaciens*-treated sample (Fig. 1b). However, analysis at the genus level also reveals a number of other distinctions between the control and the *Bacillus*-treated samples. After *Pseudomonas*, the next most abundant genera in the control samples are *Lysobacter*, *Acidovorax*, then *Rhizobium* respectively. Notably, in all *Bacillus*-treated samples, there is a marked reduction in the relative abundance of *Lysobacter* and *Acidovorax* and a concomitant increase in the relative abundance of another proteobacterium, *Acinetobacter* (Fig. 1b). The *B*. *amyloliquefaciens*-treated sample, again, shows additional unique distinctions. As well as the general decreases in relative abundance of *Lysobacter* and *Acidovorax*, and the specific, marked decrease in *Pseudomonas*, there is also a loss of *Rhizobium* and, in this sample, these changes are accompanied by an increase in the relative abundance of a wide range of genera, with a particularly dramatic increase in *Dyadobacter*, *Variovorax*, *Tahibacter*, and *Sphingomonas* (Fig. 1b). The increase in *Dyadobacter* largely accounts for the increase in *Cytophagia* previously noted at the class level, while the increase in *Variovorax* largely accounts for a dramatic increase in *Comamonadaceae*, the most notable change observed at the family level in the *B*. *amyloliquefaciens*-treated sample (Fig. 1c). *Tahibacter* falls into the family *Xanthomonadaceae*, along with *Lysobacter*. Thus, while the order *Xanthomonadales* and the family *Xanthomonadaceae* show a decrease in all other *Bacillus*-treated samples in keeping with the general decrease in *Lysobacter*, there is no such decrease in the *Xanthomonadales* or *Xanthomonadaceae* observed for the *B*. *amyloliquefaciens*-treated sample (Fig. 1c, d). The increase in *Sphingomonas* as a result of *B*. *amyloliquefaciens* treatment is not, however, unique. An increase in *Sphingomonas* is also observed in the *B*. *subtilis*-treated sample and to some extent in the mixed *Bacillus* spp.-treated sample, forming the only other major treatment-specific changes in the endophytic community observed in the assay. Other than the increase in *Sphingomonas*, though, the specific changes in the *B*. *amyloliquefaciens*-treated sample were not observed in mixed *Bacillus* spp.-treated sample (Fig. 1).

It is also notable at the genus level that the genus *Bacillus* comprised a very small proportion of the endophytic bacterial community in all samples and there was no clear change in the relative abundance of *Bacillus* between control and treated groups (Fig. 1b), suggesting that, despite the effects of external *Bacillus* inoculation on the endophytic community in sprouting broccoli, *Bacillus* is not, itself, an important part of that community.

**Fig. 2:**
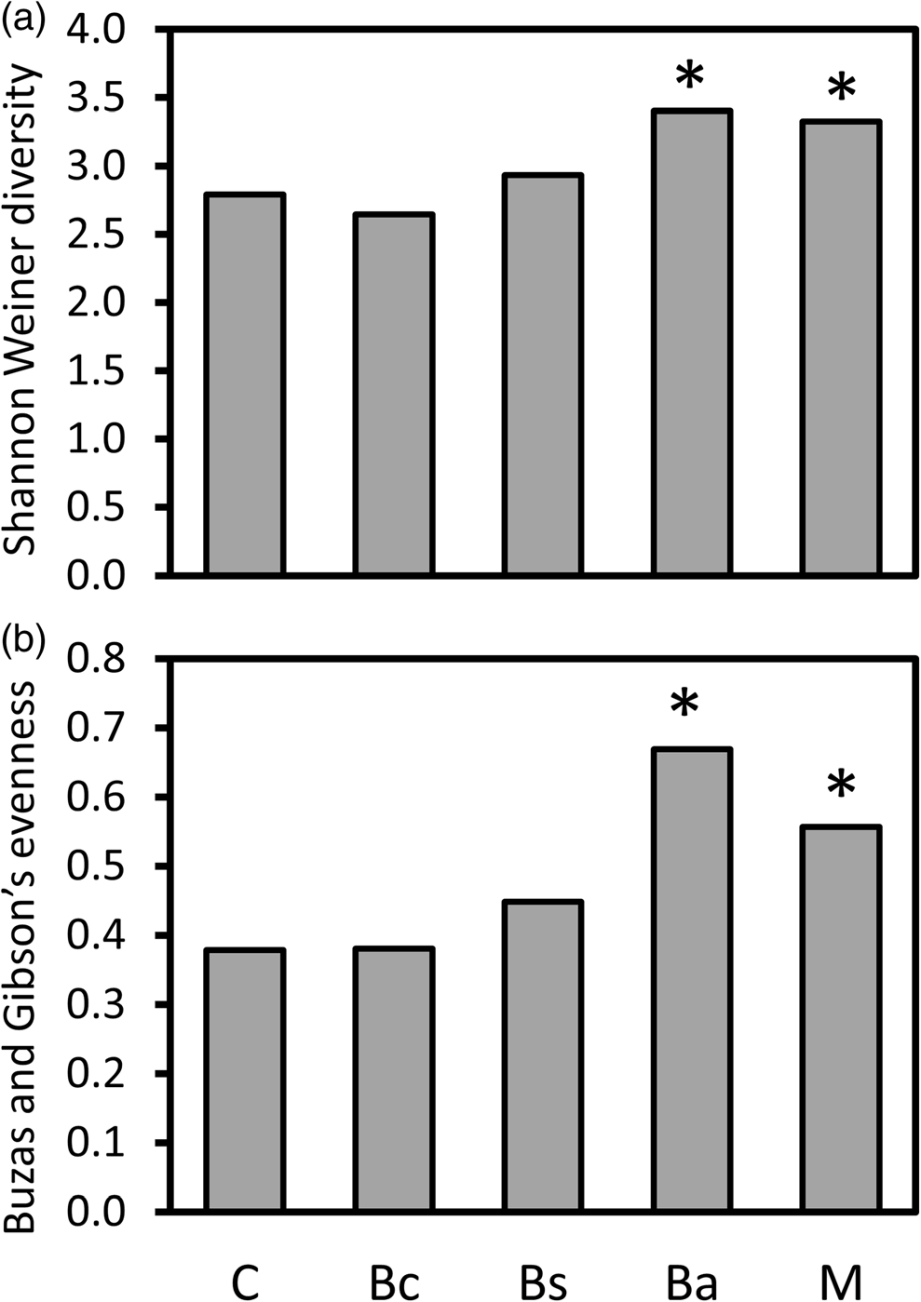
The variation in ecological measures. **a** Shannon-Weiner diversity. **b** Buzas and Gibson’s species evenness in control and treated sprouting broccoli plants. The notations B. c., B. s., B. a. represent *B*. *cereus*, *B*. *subtilis*, and *B*. *amyloliquefaciens* respectively. Asterisks represent significant differences from control samples at *p* < 0.05 based the PAST3 Diversity *t* test function

An analysis of alpha diversity via the Shannon-Weiner diversity index following sample rarefaction also revealed a marked effect of external *B*. *amyloliquefaciens* supplementation on the endophytic bacterial community. Inoculation with *B*. *amyloliquefaciens* resulted in a significant increase in the Shannon-Weiner diversity index versus that of the control sample (*p* = 5.23 × 10^−7^ based on a *z* test) (Fig. 2a). Samples treated with *B*. *cereus* or *B*. *subtilis* showed no significant change in Shannon-Weiner diversity index versus those with control plants (*p* = 0.50 and *p* = 0.14 respectively). However, treatment with the mixture of all three *Bacillus* spp. resulted in a significant increase in diversity (*p* = 2.52 × 10^−3^), similar to that observed for addition of *B*. *amyloliquefaciens* alone (Fig. 2a).

Likewise, Buzas and Gibson’s evenness index showed no significant difference when comparing control samples with *B*. *cereus-* or *B*. *subtilis*-treated samples (*p* = 0.88 and *p* = 0.10 respectively) (Fig. 2b). However, *B*. *amyloliquefaciens* (*p* = 1.21 × 10^−10^)- and mixed *Bacillus* (*p* = 1.81 × 10^−10^)-treated samples showed significantly higher evenness than the control sample (Fig. 2b). Thus, as well as causing specific changes in the composition of the endophytic bacterial community when added as a single inoculant, *B*. *amyloliquefaciens* significantly altered the wider structure of the endophytic bacterial community when added either as a single inoculant or as part of a mixed *Bacillus* culture.

**Fig. 3:**
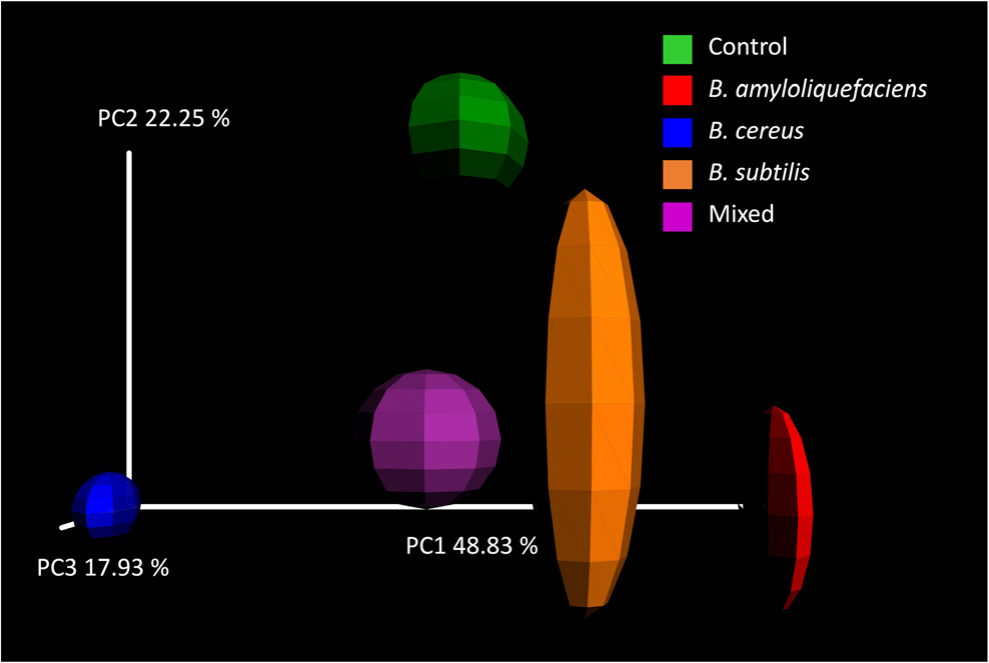
PCoA plot representing the weighted UniFrac beta diversity distance matrix across different treatments, *B*. *cereus*, *B*. *subtilis*, and *B*. *amyloliquefaciens*. Ellipses represent jackknifed estimates of confidence

Beta diversity was portrayed using the weighted (quantitative) UniFrac distance measure [34], which is a phylogenetically aware measure of beta diversity. The weighted UniFrac distance matrix was represented in a three-dimensional PCoA plot which allowed the dimensionality of the matrix to be reduced so that the most important orthogonal sources of variation between the samples could be denoted as the first, second, and third principal coordinates. The PCoA analysis indicated that the first three principal coordinates accounted for 48.83, 22.25, and 17.93% of the variation within the matrix, respectively. Thus, the first three principal coordinates account for over 89% of the variation in the UniFrac distance matrix and form a very good representation of that matrix. This analysis demonstrated significant differences in the endophytic bacterial community between all samples based on lack of any overlap between 95% confidence ellipsoids for the UniFrac beta diversity estimates plotted on the PCoA plot (Fig. 3). However, along the first principal coordinate axis, revealing the greatest source of difference between the samples, there is a clear overlap between the 95% confidence ellipsoids of control and mixed *Bacillus*-treated samples with the *B*. *subtilis*-treated sample also falling very close to these samples around the midpoint of this axis. The *B*. *amyloliquefaciens*-treated sample holds a distinct position at one end of this axis while the *B*. *cereus*-treated sample holds a distinct position at the other (Fig. 3). Possibly, the intermediate position of the mixed *Bacillus*-treated sample among the first principal coordinate represents the canceling out of opposing effects of *B*. *amyloliquefaciens* and *B*. *cereus* treatment. Consistent with this proposal, *B*. *cereus* treatment resulted in an increase in the relative abundance of the most abundant genus, *Pseudomonas*, and a decrease in the relative abundance of minor taxa as opposed to the decrease in relative abundance of *Pseudomonas* and an increase in the relative abundance of minor taxa seen for *B*. *amyloliquefaciens* treatment (Fig. 1b). The fact that the second principal coordinate separates the control sample from the *B*. *amyloliquefaciens*-, *B*. *cereus*- and mixed *Bacillus*-treated samples (Fig. 3) also confirms that all three of these regimes significantly altered the bacterial endophyte community.

## Discussion

We studied the effects of external application of individual and mixed *Bacillus* species on the sprouting broccoli root endophytic bacterial community using 454 pyrosequencing. Since over 99% of bacteria in a range of environmental samples have been shown to be unculturable [36], we took a culture independent approach, wherein total bacterial DNA from each sample was extracted and 16S rRNA amplicons were sequenced to analyze what is in effect the entire root bacterial communities from differentially treated and untreated plants. Primers equivalent to those chosen have previously been shown to amplify over 90% of all species tested and to show high coverage of almost all phyla [37]. We found that the addition of these ubiquitous bacterial species to plants changed the diversity, evenness, and composition of their indigenous endophytic bacterial communities, in a context-specific manner, upholding our original hypothesis.

The relative abundance of the genus *Bacillus* itself, however, was very low in all plants despite its external addition to seeds and soil samples. *Bacillus* is one of the most abundant bacteria in the soil [38] and rhizosphere [22]; however, despite its ubiquitous nature and two external applications, its abundance was comparatively lower than that of the majority of predominant endophytic bacteria. *Bacillus* has been found to be a significant contributor to the endosphere within many other species such as rice, maize, potato, grapevine, coffee, and coconut [11]. It is important to note that this observation may be specific to the soil type used in this study but our observations are similar to those in the related plant species, *Brassica napus*, where *Bacillus* was only found in low relative abundance in most cultivars [39]. In contrast, another common endophyte, *Pseudomonas* [35], was the dominant genus in all samples and this also mirroring the situation found in *B*. *napus* [39]. Apart from *Pseudomonas*, the predominant genera in the sprouting broccoli endosphere were *Lysobacter*, *Acidovorax*, and *Rhizobium*, again, all commonly observed endosphere genera [40, 41]. This lack of recruitment of the added *Bacillus* inocula to the endosphere suggests that the *Bacillus* species may have instead exerted their effect on endophyte recruitment in the rhizosphere, most likely via competition with other rhizosphere bacteria. However, it is critical to note that the overall structure and composition of microbial community, plant-associated factors, and environmental conditions may have shaped the outcome of *Bacillus*-mediated effects on bacterial endophytes. A thorough investigations analyzing most of these components in real time would provide a comprehensive understanding of these intricate interactions.

There were a number of common effects of *Bacillus* supplementation on endosphere composition, notably, a marked reduction in the relative abundance of *Lysobacter* and *Acidovorax*, perhaps suggesting a general competition between *Bacillus* spp. and these genera possibly through antagonism in earlier stages when the majority of bacteria colonize roots [25]. The competition for nutrients and niches may have prevented several other genera from colonizing germinating seeds and seedlings. On the other hand, there was an increase in the relative abundance of *Acinetobacter*. It may, therefore, be that *Acinetobacter* was previously inhibited due to antagonistic activities of *Lysobacter* and *Acidovorax*. However, the most dramatic effects were species specific. Addition of *B*. *amyloliquefaciens* resulted in a large decrease in the relative abundance of the most common endophyte, *Pseudomonas*. There was also a loss of *Rhizobium*, with these changes being accompanied by an increase in the relative abundance of a wide range of genera, particularly *Dyadobacter*, *Variovorax*, *Tahibacter*, and *Sphingomonas*. This outcome suggests a significant impact of *B*. *amyloliquefaciens* on the bacterial community within the rhizosphere from which the endosphere community is recruited, while again suggesting that the genera *Dyadobacter*, *Variovorax*, *Tahibacter*, and *Sphingomonas* may have previously failed to proliferate within roots, possibly due to antagonistic activities of *Pseudomonas* and *Rhizobium*.

Apart from numerous bacterial interactions in rhizospheres and endospheres [15], a variety of extraneous factors such as root metabolites, plant growth stage, native rhizosphere microbial community [42, 43], competence for nutrients and niche, and soil type may have played important roles in determining relative abundances of different taxa in each group and thus in shaping the overall endophytic community structure and dynamics [5, 14, 44].

Curiously, the effects of *B*. *amyloliquefaciens* supplementation were not observed with the mixed inoculum which also contained *B*. *amyloliquefaciens*. Such a phenomenon has been observed in studies exploring the effects of bacterial inocula on plant resistance to pests [21, 45, 46]. Effects of single versus multiple species inocula have been proposed to depend on the abundance of the added species among the indigenous microbial community at the target site. Addition of single indigenous species may boost that species at the expense of others and it may, in fact, be this decrease in other key species which affects the outcome. Conversely, addition of multiple indigenous species will boost all of those species equally and, if this mixed inoculum now also includes the key species too, the outcome would not be observed [29]. Here, *Bacillus* is a ubiquitous component of the rhizosphere and, thus, it is very possible that several or even all of the added species are indigenous. Addition of *B*. *amyloliquefaciens* would likely boost that species at the expense of others and it may be that it is a decrease in one of the other trialed *Bacillus* species which impacts the ultimate recruitment to the endosphere. Conversely, addition of multiple *Bacillus* species will boost all equally and so the effects of reduction of whichever species was key would not be seen.

The alpha diversity and evenness indices were comparatively higher in *B*. *amyloliquefaciens*- and mixed-treated plants. Although the mixed treatment did not cause such dramatic effects on the major taxa as the *B*. *amyloliquefaciens* treatment, both resulted in greatly increased relative abundances of minor taxa. Addition of *B*. *cereus* or *B*. *subtilis* had no significant effect on alpha diversity or evenness, consistent with them showing little effect on the relative abundances of either dominant or minor taxa. However, assessment of beta diversity showed that they did actually significantly affect the specific composition of the endophytic community. Principal coordinate analysis of the weighted UniFrac distance matrix revealed that all inocula resulted in a significant change in beta diversity. The fact that all inocula result in significant changes to the endophytic bacteria emphasizes the importance of the rhizosphere in shaping the endosphere, as previously suggested [9–11]. It also emphasizes the sensitivity of the rhizosphere-endosphere relationship and the possibilities for external manipulation of rhizosphere community via addition of inocula. Along the first principal coordinate, *B*. *amyloliquefaciens* and *B*. *cereus* showed opposing directions of effect with possibly the intermediate position of the mixed *Bacillus*-treated sample representing the canceling out of these opposing effects. This supports the theory proposed by Gadhave et al. [29], referenced above, that addition of mixed species inocula may actually cause a loss of effects that would be observed due to addition of only one component of that mixture.

Since this experiment was a part of a larger study, we have previously published the results of our field experiment [28] designed in the exact same way, with identical resources, and location, but which instead studied the effects of the *Bacillus* spp. treatments on foliar-feeding insects and natural enemies. A number of strikingly similar patterns can be drawn from results from both studies. Firstly, *Bacillus* spp. showed the significant effects on various attributes of bacterial endophytes and field populations of cabbage aphid (*Brevicoryne brassicae*) in a context-specific manner. Secondly, the mixed treatment appeared to be not as effective as individual treatments in terms of its effects on endophytes and foliage feeders, suggesting the prevalence of interspecies competition within added *Bacillus* species. Consequently, aphid populations grew rapidly on both control and mixed treated plants, while not on the other individual treated ones. Lastly, *B*. *amyloliquefaciens* proved to be the most distinct of all three individual *Bacillus* treatments. For instance, the rates of parasitism of *B*. *brassicae* by the braconid wasp *Diaeretiella rapae* were significant on *B*. *cereus*- and *B*. *subtilis*-treated plants, while not on *B*. *amyloliquefaciens*-treated ones. This is possibly due to differential manipulation of the PGPR community by *B*. *amyloliquefaciens*. *Pseudomonas fluorescens*, an ubiquitous PGPR, is reported to play important roles in modulating plant volatile emission and in triggering natural enemy responses [47]. Its reduced abundance in *B*. *amyloliquefaciens* treatment in the current study could explain the non-significant effects of this treatment on parasitism by *D*. *rapae* in the field study [28]. None of the *Bacillus* spp. treatments produced significant effects on sprouting broccoli biomass. It is, therefore, possible that the pest suppression mediated by different *Bacillus* spp. was directly associated with a concomitant diversion of resources into the enhanced constitutive and induced plant defenses at the expense of biomass.

Thus, the effects of each bacterial species may be context specific, instead of being widely applicable, and may be the net effect of opposing actions or may even be governed by interspecific competition within the seed inoculum too. As such, this study emphasizes the need to examine effects of inocula on a case by case basis. The fact that the effects of rhizosphere inoculum addition on the endosphere are dependent on the context of other bacterial species in the rhizosphere further emphasizes the importance of testing in the field environment. The published effects of rhizobacteria addition on plant growth and biochemistry, and insect herbivores are likely to be determined indirectly via changes in endophytic bacterial communities through numerous bacterial interactions prior and post colonization [28]. Consistent with our findings, these effects and interactions are often unpredictable, which can lead to variable effects of inoculants on plants, herbivores, and higher trophic levels in the field [30]. A relatively lower endophytic and rhizosphere bacterial diversity in laboratory and green house experiments could be one of the major reasons behind the relatively more consistent performance of inoculants in these conditions than in the field. On the contrary, microbial inoculants often fail to show promising results in the field [30], possibly due to the inability of added inoculants to compete in the complex rhizosphere environment. A comprehensive understanding of bacterial communities in diverse soils and plants through high-throughput sequencing technologies will help develop inoculants that are better suited to local conditions. This will increase the magnitude of plant growth promotion via inclusion of better suited bacterial species in an inoculant and will help alleviate the inconsistencies in inoculant performance in different conditions. Such an increase in use will help to reduce the fertilizer and pesticide application rates and promote the use of this sustainable approach in agriculture.

## Concluding Remarks

Overall, the pyrosequencing results suggest that addition of single and multiple species of *Bacillus* to the roots of plants has various effects on endophytic bacterial communities, in which certain groups of bacteria are favored to various extents. This variation may not be immediately predictable. Furthermore, the addition of these common bacterial species leads to changes in the diversity, evenness, and relative abundances of endophytic bacterial communities. Despite the failure of *Bacillus* inocula to flourish as endophytes, application of these bacteria still affected various attributes of the endophytic bacterial community. Since endophytes are one of the crucial determinants of plant health, the changes in native bacterial communities resulting from inoculant addition will be crucial in the development of effective microbial inoculants that are favorable to plants and that favor other beneficial microbial species. Due to the presence of interspecific variation and competition in the performance of bacteria, we recommend individual tailoring of inoculants on each crop and in each geographical region to increase their reliability, consistency, and efficacy.
